# High-Quality Draft Genome Sequence of *Babesia divergens*, the Etiological Agent of Cattle and Human Babesiosis

**DOI:** 10.1128/genomeA.01194-14

**Published:** 2014-11-13

**Authors:** Isabel Cuesta, Luis M. González, Karel Estrada, Ricardo Grande, Ángel Zaballos, Cheryl A. Lobo, Jorge Barrera, Alejandro Sanchez-Flores, Estrella Montero

**Affiliations:** a Unidad de Bioinformática, Centro Nacional de Microbiología, Majadahonda, Spain; b Servicio de Parasitología, Centro Nacional de Microbiología, Majadahonda, Spain; c Unidad Universitaria de Apoyo Bioinformático, Instituto de Biotecnología de la UNAM, Morelos, México; d Unidad Universitaria de Secuenciación Masiva de DNA, Instituto de Biotecnología de la UNAM, Morelos, México; e Unidad de Secuenciación, Centro Nacional de Microbiología, Majadahonda, Spain; f Blood Borne Parasites, LFKRI, New York Blood Center, New York, New York, USA

## Abstract

*Babesia divergens* causes significant morbidity and mortality in cattle and splenectomized or immunocompromised individuals. Here, we present a 10.7-Mb high-quality draft genome of this parasite close to chromosome resolution that will enable comparative genome analyses and synteny studies among related parasites.

## GENOME ANNOUNCEMENT

*Babesia divergens* is a tick-borne, intraerythrocytic apicomplexan parasite that causes severe hemolytic anemia in cattle and recently has become a significant zoonotic human pathogen (1). Infection by blood transfusion is possible as well, as the infected erythrocytes are optimum vehicles for the parasite (2, 3). Erythrocytic invasion is a critical component of the *B. divergens* asexual life cycle, ensuring continual parasite replication in red blood cells (RBCs) (4) and producing a malaria-like disease. Despite the veterinary and zoonotic importance of this parasite, relatively little research has been carried out on *Babesia*, and many questions regarding the parasite’s biology remain unanswered. A better understanding of the species’ biology and host-parasite interactions may lead to improved control mechanisms and new trends in vaccine and antibabesial drug development. To facilitate these studies, we sequenced the genome of *B. divergens* human isolate (Rouen 1987) combining three different sequencing platforms to produce a high-quality draft genome.

Genomic DNA was isolated (5, 6) from *B. divergens* human RBC cultures (7). The genome was assembled in three stages. First, a total of 34,182,568 reads, representing 310× coverage, produced with the Illumina HiSeq 2000 platform, were assembled using ALLPATHS-LG (8). Second, 829,056 reads, representing a 23× coverage produced by 454, were assembled using Newbler v. 2.8 and adding the Illumina scaffolds as overlapping “fake” Sanger reads. Third, 109,329 PacBio reads from one SMRT cell run (P4/C2 chemistry) were used to close gaps or extend contig ends in the Illumina+454 hybrid assembly using PBJelly (9). Finally, ICORN2 (10) was used for platform-specific base error corrections. The final assembly had 10,797,556 bp in 514 scaffolds, with *N*_50_/*N*_90_ values of 1,084,746/6,917 bp, respectively. The GC content was 40%, similar to other *Babesia* genomes (11, 12). However, the estimated genome size read k-mer content (~11.5 Mbp) exceeded the observed size in other *Babesia* species and in other *B. divergens* strains (~9 Mbp) (11–13). This could be due to a different repeat content (~30%) or small contigs containing genes with several haplotypes that were not resolved by the assemblers. While this work was in progress, the genomes of *B. divergens* strains (Rouen 1987 and 1802 A strains) were published (12). The Rouen 1987 sequence was reported as a very fragmented assembly with an arbitrary scaffold ordering. In contrast, our assembly contained very large scaffolds where a high number of telomeric repeats are found, indicating reconstruction of almost complete chromosomes. Using CEGMA v. 2.5 (14), we obtained 80% completeness of the genome. Protein-coding genes (3,741) predicted using Augustus (15) and the CEGMA completion level obtained were very similar to those for the recently published *B. divergens* genome (12), although the degree of continuity obtained in our study is superior, as presented in the assembly statistics.

Therefore, the *B. divergens* genome presented here can be used as a reference to study its genome structure in order to understand the regulation of its genes and probe shared synteny with other *Apicomplexan* species.

### Nucleotide sequence accession numbers.

This genome shotgun project has been deposited at the European Nucleotide Archive under the accession numbers CCSG01000001 to CCSG01000514.
